# Case Report: A severe case of traumatic ankle fracture-dislocation with medial malleolar bony defect and 5-year follow-up

**DOI:** 10.3389/fsurg.2025.1553498

**Published:** 2025-04-14

**Authors:** Wei-Cheng Hung, Chien-Chun Chang, Chien-Chung Kuo

**Affiliations:** ^1^ Department of Orthopedics, China Medical University Hospital, Taichung, Taiwan; ^2^ Minimally Invasive Spine and Joint Center, Taichung Tzu Chi Hospital, Buddhist Tzu Chi Medical Foundation, Taichung, Taiwan; ^3^ Department of Orthopaedic, Taichung Tzu Chi Hospital, Buddhist Tzu Chi Medical Foundation, Taichung, Taiwan; ^4^ Department of Leisure Industry Management, National Chin-Yi University of Technology, Taichung, Taiwan; ^5^ Department of Orthopedics, School of Medicine, China Medical University, Taichung, Taiwan

**Keywords:** ankle fracture, bone graft, deltoid ligament, flap, medial malleolar bone loss

## Abstract

Open fracture-dislocations with significant malleolar bone and soft tissue loss, along with ligament injuries, are complex and often require extensive treatment and staged surgeries. The aim of this report is to highlight treatment strategies and key considerations, including surgical reconstruction with allografts or autografts and various flap techniques as viable options. Additionally, deltoid ligament repair or reconstruction is crucial for surgical success. We report the case of a 29-year-old woman with no significant medical history who sustained an open fracture-dislocation of the left medial malleolus and midfoot due to a lifeboat propeller injury while white water rafting. The injury led to substantial bone and soft tissue loss and deltoid ligament injury. Following multiple wound debridements, the ankle was successfully reconstructed using an AO distal tibia plate, autogenous iliac bone graft, tension band wire, cancellous screw, and free anterolateral thigh flap, with direct deltoid ligament repair. The patient was followed up for 5 years, with favorable outcomes.

## 1 Introduction

Common treatments for intra-articular tibial pilon fractures include open reduction and internal fixation, minimally invasive plate osteosynthesis, and external fixation. Clinical outcomes are influenced by soft tissue conditions and joint anatomical reconstruction (1). However, open ankle fractures with concomitant bone and soft tissue loss, particularly involving the malleolar bone, are rare. The ankle joint features a saddle-shaped articulation involving the talus, tibia, and fibula, with its stability reliant on the integrity of the medial and lateral osseoligamentous complexes and distal tibiofibular syndesmosis. Reconstruction of severe open ankle injuries often requires removing infected foreign bodies, aggressive wound excision, and vascularized soft tissue coverage, which can effectively restore the ankle to a condition similar to its preinjury state (2).

Severe open ankle injuries with medial malleolus (MM) loss, albeit rare, can significantly compromise ankle stability and predispose patients to recurrent instability (3). While the MM contributes minimally to ankle stability in the neutral position, its role becomes more pronounced under varus and valgus stress, accounting for up to 22% of stability (4). Previous literature has utilized various treatment approaches to manage bone loss, ligament injuries, and soft tissue defects with different flap techniques. However, there is a lack of mid-to-long-term follow-up studies and well-established postoperative rehabilitation protocols. This case report describes the reconstruction of the left ankle using an iliac crest graft, deltoid ligament repair, and an anterolateral thigh flap to address traumatic MM loss, resulting in a favorable functional outcomes and sustained range of motion (ROM) even after 5 years post-injury.

The patient provided informed consent to the publication of her data related to this case report.

## 2 Case report

**Figure 1:**
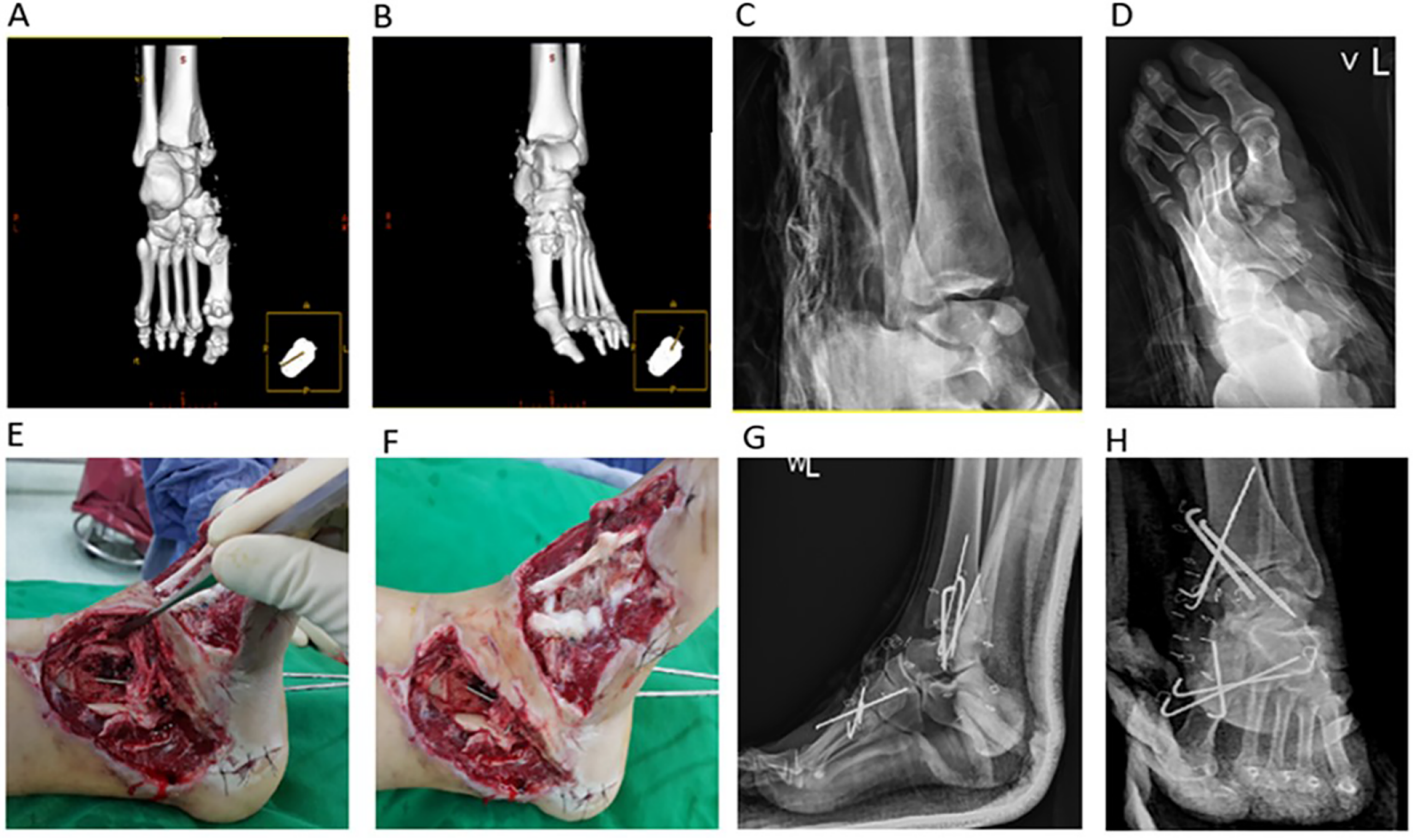
**(A–D)** Preoperative images **(E,F)** intraoperative images **(G,H)** postoperative images. Initial radiographs and CT-based 3D bone reconstruction of the left ankle show a large bony defect at the MM and midfoot fractures **(A–D)**. During surgery, devitalized tissues were removed, thereby revealing significant bone loss, deltoid ligament injury, and soft tissue defects in the MM and foot. The MM remnant was stabilized with two 1.8 mm Kirschner wires. Two 2.0 mm Kirschner wires were used to address the tibiotalar dislocation **(E,F)**. Postoperative radiographs show two 1.8 mm Kirschner wires stabilizing the MM, three 1.8 mm Kirschner wires for the 1st and 2nd tarsometatarsal joints (i.e., Lisfranc injury), and two 2.0 mm Kirschner wires for the tibiotalar dislocation **(G,H)**. CT, computed tomography; 3D, three-dimensional; MM, medial malleolus.

A 29-year-old woman with no significant medical history sustained a left ankle injury on August 9, 2018, when her left ankle was accidentally lacerated by a lifeboat propeller while whitewater rafting in Hualien, Taiwan. She was transferred from a local hospital, where examination and imaging revealed a Gustilo–Anderson type IIIB open fracture-dislocation with bone loss, deltoid ligament injury, and soft tissue loss (preoperative images: Figures 1A–D). The posterior tibial and dorsalis pedis arteries were palpable, and no other significant injuries were found.

Her injuries included fractures of the left medial navicular bone, left 1st and 2nd metatarsal bases, and left medial and middle cuneiform bones, along with Lisfranc injuries. Within 24 h of admission, she underwent wound debridement to remove devitalized tissue and foreign bodies. Notably, the injury involved a 2.5 cm × 2.5 cm pyramidal-shaped bone loss with deltoid ligament injury and a 7 cm × 8 cm soft tissue defect over the left MM.

Two 1.8 mm Kirschner wires were used to stabilize the MM bone defect; three 1.8 mm Kirschner wires to stabilize the 1st and 2nd tarsometatarsal joint injury (i.e., Lisfranc injury); and two 2.0 mm Kirschner wires to stabilize the tibiotalar joint dislocation (postoperation images: Figures 1G,H). Negative-pressure wound therapy was applied to the open wound at −125 mmHg. On August 20, 2018, the patient was referred to China Medical University Hospital (Taichung, Taiwan, ROC) for further treatment.

### 2.1 Wound presentation

The patient presented with a 7 cm × 8 cm laceration with skin and soft tissue loss on the medial left ankle. She exhibited medial distal tibial and dorsal navicular bone loss, along with tibiotalar joint and deltoid ligament injuries. The ankle, subtalar, talonavicular, naviculocuneiform, and 1st and 2nd tarsometatarsal joints were exposed, with superficial bone shavings in all affected areas (Figures 1E,F).

### 2.2 Surgical technique

Debridement and negative-pressure wound therapy were performed twice before reconstruction. A combined orthopedic and plastic surgery procedure was performed 10 days later for definitive management.

**Figure 2:**
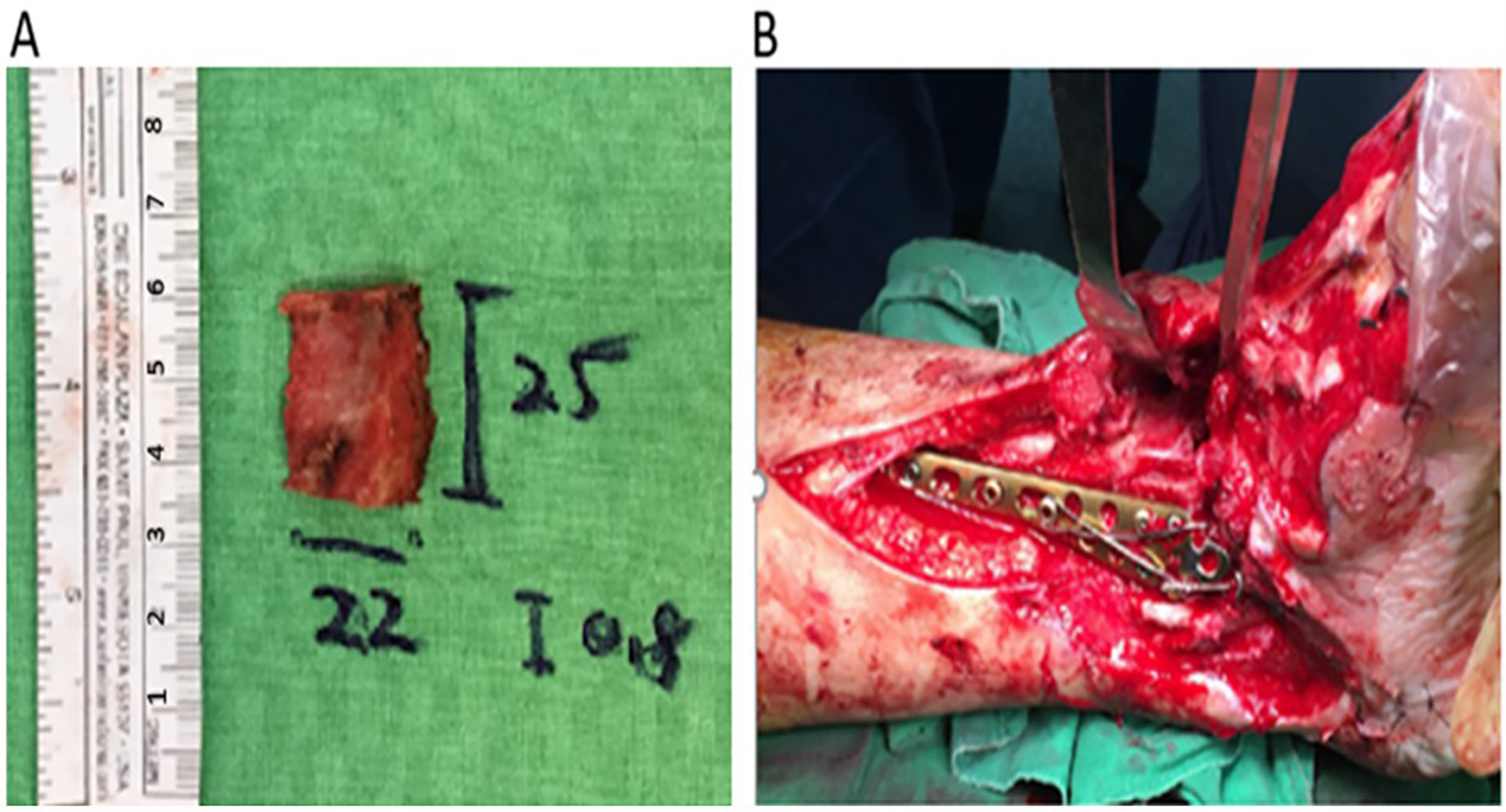
Intraoperative images of the final reconstruction **(A)** A slightly oversized bone graft (2.5 cm × 2.2 cm × 0.8 cm) is harvested from the inner pelvis, 2.5 cm behind the anterior superior iliac spine. The graft was trimmed to fit the MM contour, and an AO distal tibial plate was used to secure it. **(B)** A tension band wire and two 1.5 mm Kirschner wires have been added to improve graft stability after plate fixation. MM, medial malleolus.

**Figure 3:**
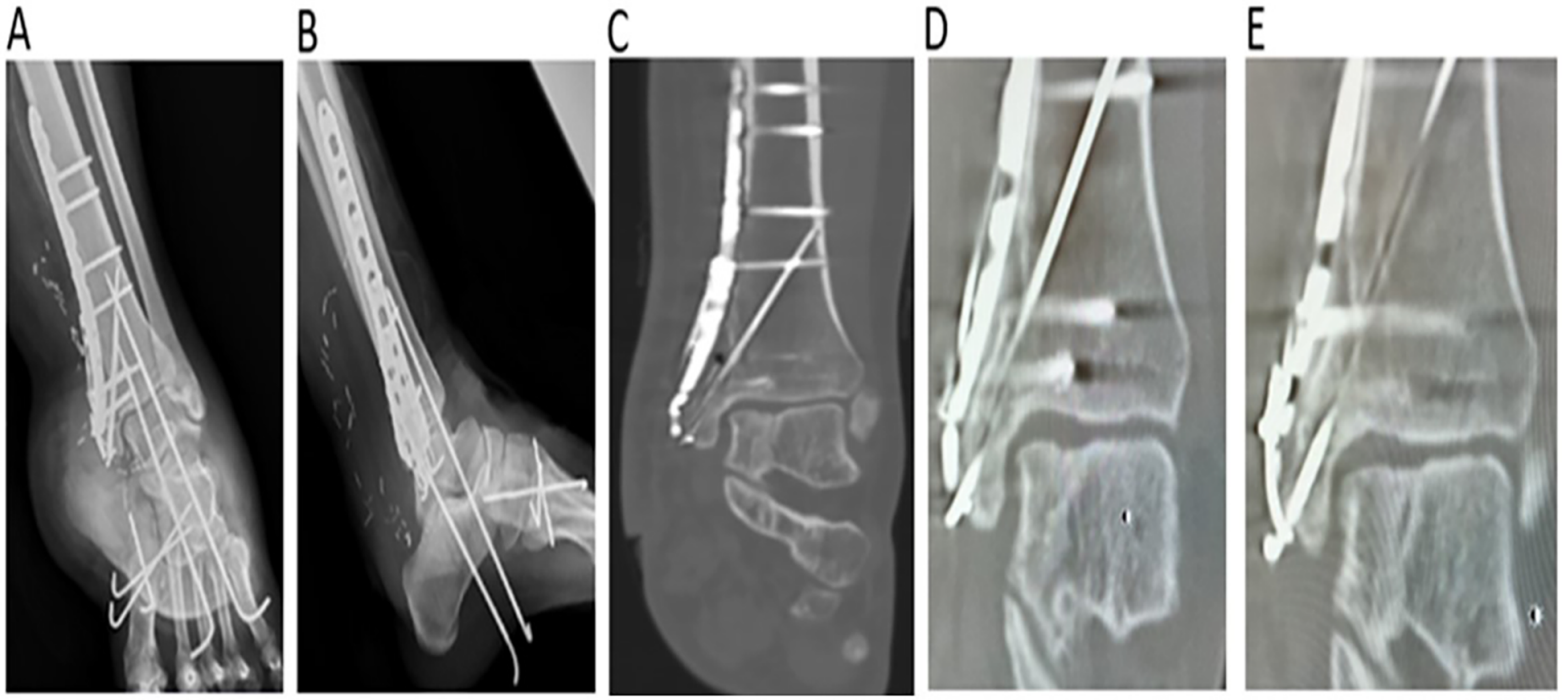
**(A,B)** Radiography findings postoperation **(C)** CT imaging findings at 2 years postoperation **(D,E)** CT imaging findings at 5 years postoperation. Radiographs of the right ankle show proper alignment at the MM reconstruction site and appropriate ankle joint space **(A,B)**. Postoperative CT scans at 2 **(C)** and 5 years **(D)** show good bone consolidation without bone absorption at the MM reconstruction site and appropriate joint space. CT, computed tomography; MM, medial malleolus.

The MM was reconstructed using a bicortical iliac bone graft from the left anterior superior iliac spine (Figure 2A). A slightly oversized bone graft was obtained 2.5 cm posterior to the anterior superior iliac spine from the inner pelvic table and shaped with a rongeur and bone file to mimic the distal tibial curvature. Before applying the internal fixation device, the injured deltoid ligament was identified and repaired using 2-0 Vicryl sutures (Ethicon, Somerville, NJ, USA). An AO distal tibial plate, tension band wire, and cancellous screw were used to fix the iliac bone graft to the MM (Figure 2B). Postoperative imaging confirmed proper alignment, with the bone graft securely fixed to the bony defect using an AO distal tibial plate and tension band wire (Figures 3A,B).

A plastic surgeon performed soft tissue coverage using a free left anterolateral thigh flap (8 cm × 28 cm) for plate coverage. The descending branches of the lateral femoral circumflex artery and vein were anastomosed to the posterior tibial artery and vein above the injury site. The donor site and the remaining soft tissue loss at the recipient site were covered with a meshed split-thickness skin graft. After 6 days, a split-thickness skin graft from the scalp was applied to the thigh flap, which was monitored for 2 weeks without complications.

Postoperatively, the patient wore a non-weight-bearing (NWB) short-leg splint for 6 months and initiated active ROM exercises after 1 month. At the 3-month follow-up, the ankle joint demonstrated stability with satisfactory bone healing and soft tissue contours over the MM. The patient's weight-bearing status was subsequently upgraded to partial weight bearing. By 6 months post-operatively, she achieved fully-weight bearing capacity and returned to work, with no donor site complications in the left thigh or pelvis.

### 2.3 Clinical outcomes

**Figure 4:**
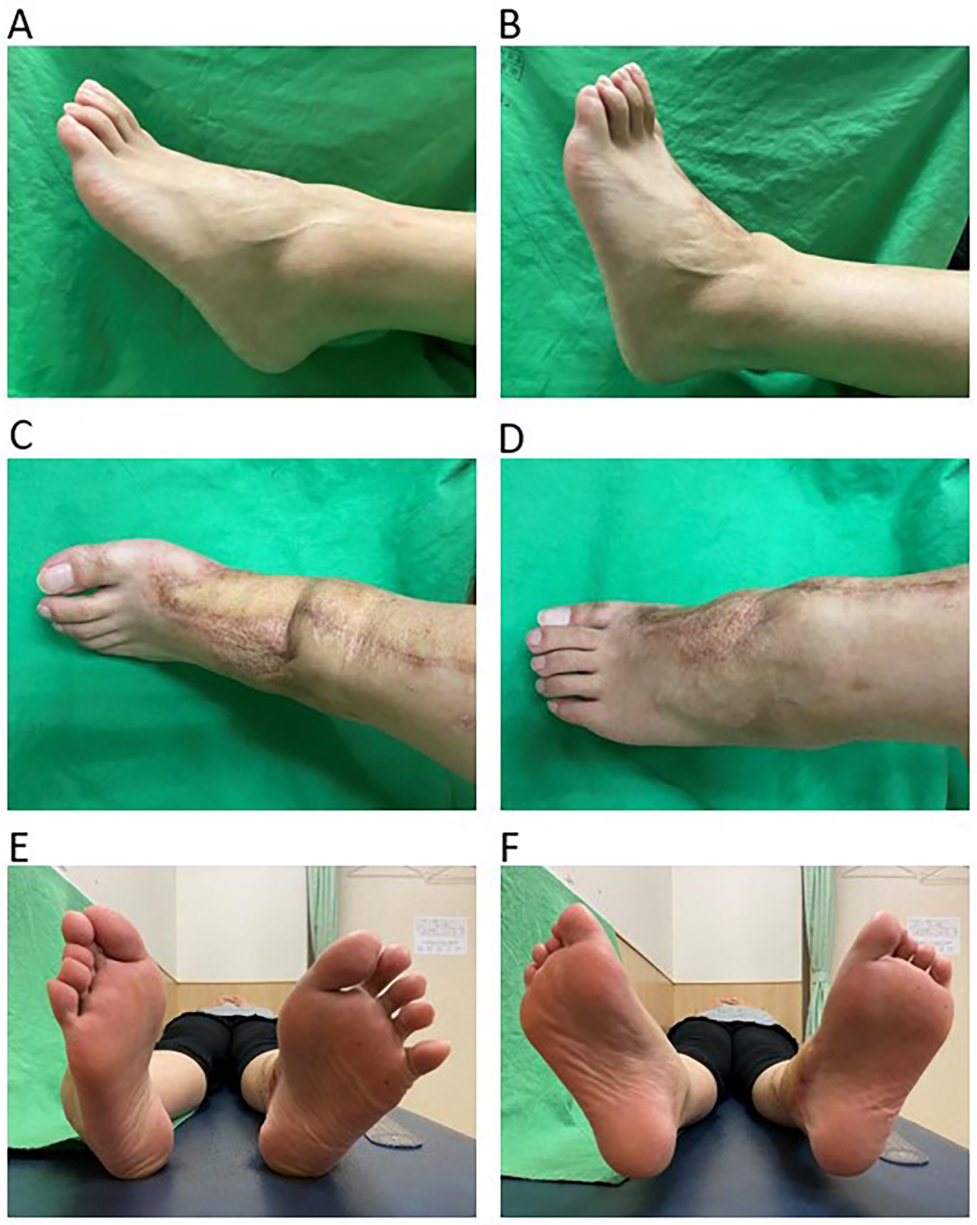
The images show the gross views of ROM. Plantarflexion **(A)** and dorsiflexion **(B)** of the left ankle after 5 years of ankle ROM follow-up. The dorsal view shows inversion **(C)** and eversion **(D)** of the left foot. The plantar view shows inversion **(E)** and eversion **(F)** of both feet. ROM, range of motion; MM, medial malleolus.

The patient's postoperative range of motion (ROM) progressively improved. At week 2, passive dorsiflexion and plantarflexion were limited to 0–5° and 5–10°, respectively. By week 4, active dorsiflexion and plantarflexion reached 0–5° and 10–15°. At month 3, they improved to 10° and 25–40°, with mild eversion and inversion recovery (10–15° and 15–20°). By month 6, ROM remained unchanged. At year 1, dorsiflexion and plantarflexion reached 10° and 35–45°, while eversion and inversion improved to 15° and 20–25°. By year 2, dorsiflexion remained at 10°, plantarflexion at 35–45°, and eversion/inversion at 15°/20–25°. At year 5, near-normal ROM was achieved, with dorsiflexion at 15°, plantarflexion at 45°, and eversion/inversion at 15°/30° (Figures 4A–F). The left ankle exhibited favorable ROM recovery.

The results of computed tomography scans follow-up at 2 years and 5 years post-operatively demonstrated good bony consolidation at the MM reconstruction site without evidence of joint space narrowing (Figures 3C–E).

The evaluation of the American Orthopedic Foot and Ankle Society (AOFAS) score at 60 months was described as follows: no pain was noted, and functional assessment revealed no limitations, no need for support, a maximum walking distance of more than six blocks, and no difficulty walking on any surface. There was no gait abnormality, sagittal motion was normal, and ankle-hindfoot stability was well maintained. However, some degree of ankle-hindfoot malalignment was observed without symptoms. The total score was 95 points.

### 2.4 Timeline

**2018/8/9:** Left ankle was accidentally lacerated by a lifeboat propeller

**2018/8/9-8/20:** Debridement with k-wire temporary fixation and negative-pressure wound therapy

**2018/8/20:** Referred to China Medical University Hospital

**2018/8/20-9/3:** Debridement and negative-pressure wound therapy were performed twice

**2018/9/3:** Deltoid ligament repair, MM reconstruction with ASIS autograft and ORIF with AO distal tibial plate, tension band wire and cancellous screws

**2018/9/13:** Soft tissue reconstruction with free left anterolateral thigh flap

**2018/9/13-2019/3/15:** NWB short-leg splint for 6 months

**2019/3/15 ∼:** Active ROM exercises

## 3 Discussion

In this case report, we described the reconstruction of the left ankle using an iliac crest graft, deltoid ligament repair, and an anterolateral thigh flap to address traumatic MM loss. Postoperatively, the patient exhibited good functional outcomes and ROM, which were maintained after 5 years of follow-up. The ankle typically bears up to four times a person's body weight (4). In the neutral position, 80%–90% of the body weight is transmitted through the tibiotalar joint, with the rest borne by the medial and lateral malleolar joints (5).

Complicated cases of severe ankle trauma may require extensive treatment and staged surgeries. This report presents a successful MM reconstruction after severe traumatic loss. In managing complex ankle fractures, a comprehensive treatment approach is crucial to ensure joint stability and mitigate infection risks associated with soft tissue defects (6). The treatment principles involve debridement, managing ligament injuries and bone defects, restoring alignment, addressing soft tissue defects, and rehabilitation, all of which are essential for optimal outcomes.

Regarding the management of ligament injuries, in cases of MM bone loss, the deltoid ligament is primarily affected (7). The deltoid ligament comprises deep and superficial components that attach to the MM, talus, calcaneus, and navicular bones. It plays a key role in restraining external rotation and eversion of the talus, relative to the tibia (8). Stabilizing the talus, subtalar joint, and medial foot arch is essential. Chronic instability, degeneration, and increased joint pressure have been linked to the deltoid ligament, as demonstrated in biomechanical studies (9). Proper repair is crucial to reduce instability, protect the cartilage, and slow the progression of ankle osteoarthritis (7, 10, 11). Repair is primarily categorized into two types: direct suture repair for midsubstance ruptures and single-suture anchoring into the fibula for fibular avulsions (8, 12, 13). In our patient, the rupture was a superficial tear rather than an avulsion. Therefore, we used the single-suture technique for the repair.

Syndesmosis injury is a potential complication of severe ankle fractures. Current treatment modalities include dynamic fixation with a suture button and static fixation with a trans-syndesmotic screw. Although no consensus exists for the definitive optimal treatment, biomechanical studies have shown that using a suture button can prevent abnormal motion of the tibiofibular joint and achieve proper reduction (14). In this case, intraoperative examination and imaging did not reveal any significant syndesmosis injury during.

The management of traumatic bone defects is influenced by various factors, including defect size, comorbidities, soft tissue conditions, and infection risk. With multiple available treatment options, selecting the most effective approach is crucial for achieving optimal outcomes. Bone grafting is generally preferred for small defects, while larger defects may require more advanced techniques, such as induced membrane technique or distraction osteogenesis (15–17).

Allografts and autografts are the primary graft options for small bone defects. Autogenous bone grafts are the gold standard treatment for posttraumatic conditions. Cortical autografts provide strong mechanical support and can be harvested with or without vascular supply (18). In our patient, we used a cortical autologous bone graft by harvesting an iliac bone graft of similar size and shaping its medial aspect to mimic the curvature of the distal tibia and the pyramidal-shaped bony defect over the MM.

Suitable plates and allografts are employed to achieve proper alignment at the fracture site. Nithyananth et al. (19) successfully reconstructed a traumatic MM with bone loss using an iliac bone graft and cancellous screws. However, this approach was not feasible in our patient due to the small size of the remaining MM. Kow et al. (20) reconstructed a traumatic MM with bone loss using an iliac bone graft fixed with a modified low-profile nine-hole one-third tubular plate, acting as an antiglide plate. A biomechanical study by Wegner et al. (21) demonstrated that an antiglide fixation construct was stiffer and could withstand higher loads before failure than constructs using bicortical, divergent unicortical, or parallel unicortical screws for vertical MM fractures. In our patient, ankle instability worsened because of the loss of the MM, ankle dislocation, injured deltoid ligament, and soft tissue loss. Open fixation with an image intensifier was performed to ensure proper tibial plateau reconstruction. The bone graft was securely fixed to the MM using an AO distal tibial plate as the antiglide plate, along with a tension band wire and cancellous screws.

Regarding soft tissue coverage, Nithyananth et al. (19) utilized a reverse sural flap, whereas Kow et al. (20) used a left radial forearm free flap for soft tissue coverage. Free flaps are the gold standard for covering lower leg wounds as they effectively address large defects with high success rates and can be used in acute situations with distant recipient vessels (19, 20, 22). Bajantri et al. (23) recommend using a gracilis muscle flap for small-to-medium defects, a latissimus dorsi muscle flap for large defects, and an anterolateral thigh flap for skin coverage. The free anterolateral thigh flap is particularly advantageous in foot and ankle reconstruction because it covers large defects with a high success rate (22, 24–26). In our patient, we successfully used this flap to treat large soft tissue defects and achieved favorable outcome.

A good postoperative rehabilitation program is critical for successful surgical outcomes. Swart et al. (27) classified patients into three clinical scenarios, based on the type of foot fracture and the patient's age, to determine the duration of the NWB status. Other factors, such as injury type, bone quality, osteoporosis, and comorbidities, were also considered when adjusting for NWB duration. Among patients aged 20–30 years with good overall health, the NWB status typically lasts for 4–6 weeks (27). Our patient was a 29-year-old woman with no significant past medical history who sustained a complicated ankle open fracture-dislocation. As a result, we implemented a more conservative protocol for NWB duration, extending it to 8 weeks, compared with the usual 4–6 weeks for ankle injuries. Partial weight-bearing was permitted only after 8 weeks, following satisfactory radiographic evidence of bone healing and flap recovery, to prevent nonunion. Active ROM exercises were initiated postoperatively once the patient tolerated the ankle stiffness.

Concomitant foot injuries can cause foot stiffness and tendon or ligament damage, thereby affecting soft tissue balance, leading to the loss of dorsiflexion. However, in our patient, active and passive ROM improved with no significant impact on the patient's daily life. Nevertheless, the ankle must be monitored for post-traumatic degenerative arthritis during long-term follow-up. Owing to the severity of the initial injury and the complexity of reconstruction, patients should be informed of this future risk.

Severe ankle trauma is associated with a higher incidence of arthritis, especially in cases of intra-articular fractures and significant ligament injuries (28). Treatment options for ankle arthritis, depending on the severity, include conservative treatment and surgical interventions, such as joint preservation techniques, arthrodesis, and total ankle arthroplasty. Due to advancements in surgical techniques and implant development, both the difficulty and success rate of surgeries have significantly improved. For example, the commonly performed and effective Tibiotalocalcaneal Arthrodesis can be performed using minimally invasive surgery with a retrograde intramedullary nail, achieving high fusion and low complication rates (29).

Due to advancements in modern technology, such as 3D model analysis, biomechanical applications, materials science, and artificial neural networks, there is now the ability to better analyze the loading conditions of ligaments, evaluate the application of implant materials, and determine the optimal placement of implants. These technologies provide more precise and effective solutions for improving surgical outcomes, enhancing the understanding of biomechanics, and refining the design and positioning of implants to better mimic natural tissue structures and functions (30–34). The application of these new technologies may be the direction for future research.

## 4 Conclusion

We described the successful reconstruction of the MM after severe traumatic loss with favorable medium-term functional outcomes. During hospitalization, we thoroughly assessed the patient's condition. The medical treatment included an appropriate course of antibiotics and pain management, as well as a rigorous postoperative rehabilitation process. This involved evaluating the ROM of the ankle, with careful assessment of improvements in dorsiflexion, plantarflexion, inversion, and eversion. The final results showed dorsiflexion of 15–20°, plantarflexion of 45°, eversion of 15°, and inversion of 30°. The patients achieved an American Orthopedic Foot and Ankle Society score of 95, demonstrating good ankle stability and recovery of the bone and soft tissue. Adhering to treatment principles, involving initial debridement, management of ligament injuries and bone defects, restoration of alignment and soft tissue defects, and rehabilitation, resulted in favorable functional outcomes. Key challenges post-injury included ankle instability from MM loss, injury to ligaments such as the deltoid ligament, and soft tissue coverage difficulties. Infection and persistent ankle instability are inherent risks. However, we addressed the significant bone loss with an iliac crest graft and covered the exposed ankle and foot with an anterolateral thigh flap. This approach demonstrated that primary bone grafting, followed by flap coverage, can yield good results with no graft absorption observed at the 5-year follow-up.

This case report presents the treatment principles for severe MM bone defects and medium-term follow-up function. Currently, no consensus exists on the treatment for this type of severe injury, and the literature lacks comprehensive reviews, case series studies, or medium-term prognostic data. Our study had several limitations, including the analysis of a single case and lack of long-term follow-up results. Future research should explore the classification of treatment approaches and functional outcomes through larger case series or cohort studies.

## Data Availability

The datasets presented in this study can be found in online repositories. The names of the repository/repositories and accession number(s) can be found below: https://drive.google.com/drive/folders/1KqE_4u_okL4dKrjVLqVs1k0UVa8hrV66?usp=drive_link.
